# A Morphology-Based Model to Describe the Low-Temperature Impact Behaviour of Rubber-Toughened Polypropylene

**DOI:** 10.3390/polym13132218

**Published:** 2021-07-05

**Authors:** Rudy Deblieck, Klaas Remerie, Winke Van den Fonteyne, Mark Boerakker

**Affiliations:** 1 SABIC, Technology and Innovation, 6167 RD Geleen, The Netherlands; winke.vandenfonteyne@sabic.com (W.V.d.F.); mark.boerakker@sabic.com (M.B.); 2 DSM, Materials Science, 6167 RD Geleen, The Netherlands; 3 Engineering and Technology Institute Groningen (ENTEG), Chemical Product Engineering, University of Groningen, Nijenborgh 4, 9747 AG Groningen, The Netherlands; k.remerie@home.nl

**Keywords:** critical ligament model, impact, impact copolymers, morphology, polypropylene, brittle-ductile transition, rubber content

## Abstract

The roles of the rubber particle size, the rubber particle size distribution and the constitutive behaviour of the isotactic polypropylene matrix have been studied by combining the Lazerri–Bucknall energy criterion for cavitation with the Van der Sanden–Meier–Tervoort ligament model adapted for impact conditions. It is concluded that an optimised morphology offers great potential to achieve enhanced mechanical properties with far less rubber and hence achieve a superior stiffness/toughness/processing balance.

## 1. Introduction

The toughening of polymers by blending in a rubber phase to improve their impact resistance for application in for example car bumpers is necessary to improve energy absorption and to avoid hazardous brittle fracture. The brittle/ductile transition of polymers is generally described by the Ludwik–Davidenkov–Orowan criterion [1] that simply states that a material fails in a ductile way as soon as the yield stress drops below the fracture stress. In general, this is achieved by reducing the yield stress by blending in a low modulus rubber phase, that cavitates easily, or easily delaminating hard particles, or simply voids [2]. In the present paper we aim at combining an existing model that describe the role of particle size combined with a model that describes the role of the critical interparticle ligament thickness [3].

## 2. Experimental

Data were taken from existing literature and part of the measurements used in this paper were performed at SABIC (Geleen, The Netherlands). The experimental details are given here.

### 2.1. Samples

One homopolymer (Melt Flow Index (MFI) 5.5 dg/10 min, measured at 240 °C and 2.16 g) and four rubber-toughened polypropylenes (PPs) with different rubber contents were prepared. The rubber contents were 5, 15, 25 and 32.7 wt %. To make the rubber-toughened PPs, the homopolymer was blended with a rubber-toughened PP (MFI 1.0 dg/min) containing 32.7% propylene-ethylene rubber. Granules were prepared with an extruder type ZSK 3033 at a temperature between 244 and 262 °C at a speed of 10 kg/h.

### 2.2. Impact Strength Izod–Brittle Ductile Temperature

Izod tests at a range of temperatures in order to determine the brittle ductile transition were performed with a Zwick Type 5110 impact tester according to ASTM D256. The dimensions of the specimen were 65 mm × 12.7 mm × 3.2 mm and the striking speed of the hammer was 3.5 m/s. The values of impact strength given were attained from an average of five tests.

### 2.3. Microscopy Techniques and Morphology Analysis

#### 2.3.1. Ultramicrotomy

Samples were trimmed at −120 °C parallel to the injection moulding machine direction. The blocks were then stained in a RuO_4_ solution for approximately 24 h. Thin sections with a thickness ranging from 60 to 100 nm were prepared using a Leica EM UC7 with a DIATOME Cryo knife operated at −120 °C.

#### 2.3.2. Imaging

Imaging was undertaken with a FEI Versa 3D HR-FEG- SEM operated at 15 kV.

#### 2.3.3. Particle Size—Interparticle Distance

The average particle size <*d*> is assessed by image analysis from scanning electron microscopy (SEM) images. For calculating the average interparticle distance, a monodisperse cubic stacking is assumed following Wu [4].

(1) $$ID_{}=<d>\left[k{\left(\frac{\pi }{6\varphi_{r}}\right)}^{1/3}-1\right]$$

where <*d*> is the average rubber particle diameter, *φ_r_* the rubber volume fraction, and *k* is a geometric constant, i.e., *k* = 1 for a simple cubic stacking, 2^1/3^ for a body-centered stacking and 4^1/3^ for face-centered stacking. In practice, Wu found that the simple cubic model best fits Nylon rubber blends, therefore we will also assume that *k* = 1. Because the average densities of the Ethylene Propylene Rubber (EPR) phase (~896 kg/m^3^) and the isotactic PolyPropylene (iPP) matrix, (~901 kg/m^3^) nearly coincide, the weight fraction and the volume fraction of rubber will be nearly identical. In the course of this paper, the volume fraction will be used.

## 3. The Brittle–Ductile Transition

### 3.1. Polymer Matrix Deformation Mechanisms

**Figure 1:**
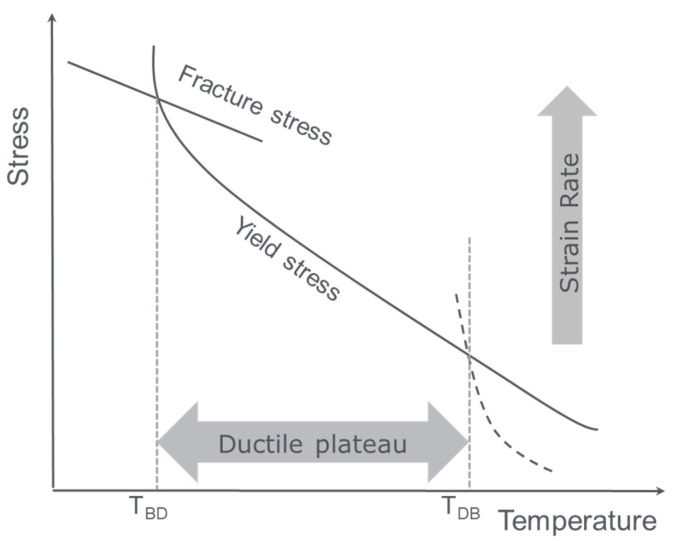
Davidenkov balance between fracture stress, yield stress, and disentanglement craze stress as a function of temperature.

Homopolymer deformation and failure mechanisms for semicrystalline polymers were already discussed in a previous publication [5]. The occurrence of these mechanisms depends on temperature and strain rate and have been observed to occur over a range of strain rates and temperatures. These include impact conditions such as in a notched Izod test, probing the brittle–ductile transition temperature. The general strain rate or temperature dependent failure response of a polymer can be plotted in a so-called Davidenkov balance [1] as shown in Figure 1, where the failure stress is plotted as a function of temperature or strain rate. In such an overview, there usually exists a temperature range or a strain rate range within which the polymer fails in a ductile way. In this ductile regime the active deformation mechanism is shear yielding characterised by large-scale plasticity. Before and beyond this ductile regime a brittle craze-crack mechanism occurs driven by either disentanglement crazing (at higher temperatures and lower strain rates) or chain scission crazing (at lower temperatures or higher strain rates such as impact).

**Figure 2:**
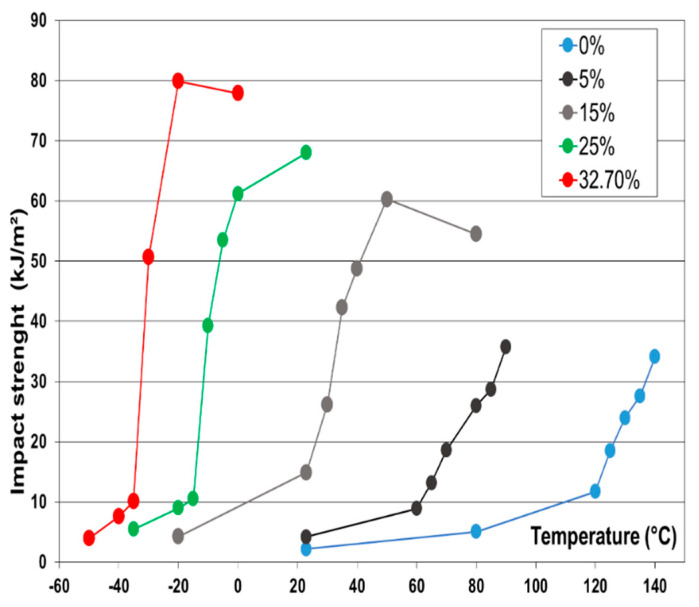
notched Izod impact data of polypropylenes with varying rubber contents over a broad temperature range. The rubber contents are indicated in the legend. The figure illustrates that the brittle–ductile transition shifts with increasing amount of rubber.

Typically, Polypropylene (PP) homopolymer under impact conditions features behaviour that is typical for the low temperature fracture regime. This is shown in Figure 2, where the notched Izod data of a PP homopolymer is compared with rubber-toughened PP grades.

It is clear that the unfilled PP homopolymer does not reach its ductile plateau before 140 °C and features brittle behaviour over the whole temperature range up to 120 °C.

Upon introduction of a low modulus, low *T_g_* (typically shear moduli ranging from 0.5–1 MPa and a glass temperature of around −60 °C) rubber phase via copolymer synthesis in a second reactor or via physical blending, the toughness increases. At rubber contents of 25 wt % and higher, the transition from brittle to ductile behaviour is observed at a temperature below −10 °C.

### 3.2. Deformation Mechanism under Impact Conditions

**Figure 3:**
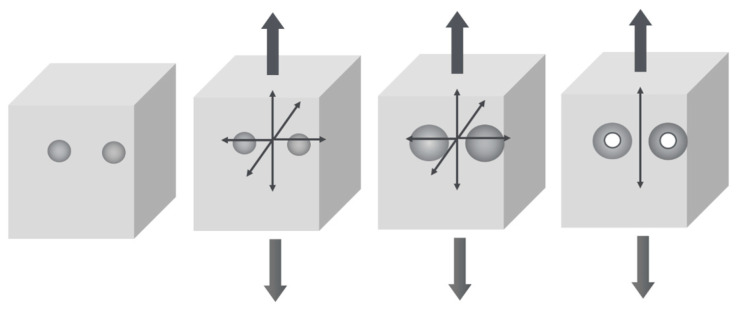
Schematic representation of the rubber cavitation mechanism and the concomitant plane strain–plane stress transition in the matrix ligament by the change from a negative hydrostatic stress state to a uniaxial stress state.

Post mortem examination of Izod test bars by means of SEM indicated damage areas that are consistent with the failure mechanism as proposed by Van der Wal and Gaymans [6]. In their description, it is suggested that the rubber particles cavitate upon deformation of the bulk phase. Consequently, the original tri-axial stress state in the matrix ligaments between the cavitated rubber particles converts into a uni-axial stress state as shown schematically in Figure 3. In this uniaxial stress situation, the yield stress is significantly reduced, leading to enhanced ductility of the ligaments, increased plasticity and hence higher dissipated energy.

**Figure 4:**
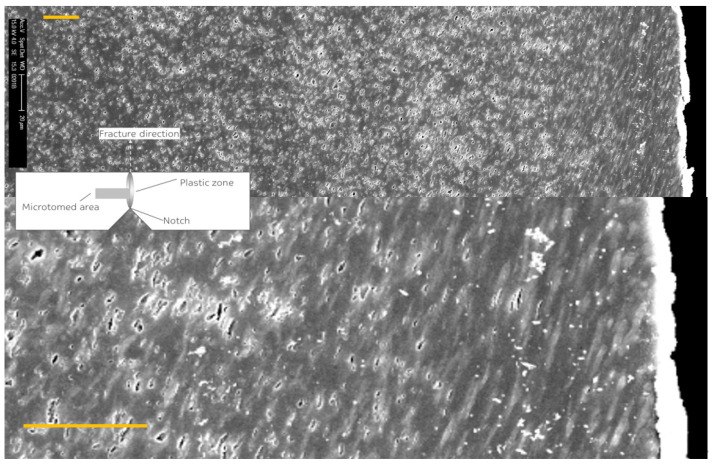
Scanning electron microscopy (SEM) micrograph of a perpendicularly microtomed and RuO_4_ stained Izod bar revealing the deformation mechanism in the plastic zone after impact failure in a typical PP impact copolymer with 30 wt % rubber. The crack surface is on the right side and crack propagation went from bottom to top. The lower image is a magnification of the crack area. The two white lines represent 20 μm.

This is shown in Figure 4 where a SEM micrograph reveals the deformation mechanism in the plastic zone of a post mortem perpendicularly cryomicrotomed and RuO_4_ stained Izod test bar. The crack surface is on the right hand side and the crack propagated from the bottom up. The horizontal dimension then represents the depth into the specimen and the plastic zone.

It can clearly be seen that extensive rubber particle cavitation occurs. The cavitation also becomes less extensive with increasing depth into the plastic zone, where the stress has dropped below the matrix yield stress. It can also be seen that around the crack propagation zone there are no cavitated rubber particles in the first 20 μm which leads us to the assumption that the cavitated particles have healed due to adiabatic temperature rise after deformation. In the first 100 to 150 μm all rubber particles are elongated in the average main Von Mises stress direction.

Experimental support for this concept has been provided by A. Van der Wal et al., who investigated the influence of particle size [7], iPP matrix crystallinity [8], rubber content [9], molecular mass and temperature of the iPP matrix [10]. In the course of this paper, the experimental data generated by these authors will be used to validate a novel description of the deformation behaviour at impact testing conditions.

## 4. The Model Description of Impact Toughness

In a number of steps, the most important features of the deformation mechanism under impact conditions will be described and connected. It is assumed that the rubber particle size distribution (PSD) is monodisperse. The ligament sizes are calculated using Wu’s regular cubic stacking equation, thereby assuming that all ligament sizes are equal. The cavitation will be described by the Lazerri–Bucknall energy criterion, whereas the response of the ligament will be described by means of the model first described by Van der Sanden, Meijer and Tervoort (VMT) in 1993 [11]. In order for this model to describe impact, the VMT equation, which describes the energy balance at failure of a ligament, will have to be adapted to the constitutive behavior at high strain rates and low temperatures.

### 4.1. The Role and Determination of Surface Energy

Both in the Lazerri–Bucknall energy balance description of the cavitation of the disperse rubber particles and in the description of the fracture strength of the matrix ligaments in the VMT model the surface energy Γ appears. Following the original definition of surface energy by Kramer and Berger [12,13]:

(2) $$\Gamma =\gamma +\frac{1}{4}\upsilon_{e}d_{e}U_{b}=\gamma +\frac{1}{2}\Sigma_{e}^{}U_{b}$$

where *γ* is the Van der Waals surface energy contribution, *U_b_* is the strength of the covalent C–C bond (i.e., 346 kJ/m^2^) and $\nu_{e}$ is the node density of load bearing chains at the time scale and the temperature of the experiment, where the suffix e stands for effective. *d_e_* is the point-to-point node distance measured along the chain which is related to *v_e_* via the number of Kuhn segments *n_L_* with length *l_k_* and mass *m_K_* in a chain segment.

(3) $$d_{e}^{}=\sqrt{n_{{}_{L}}}l_{k}^{}=\sqrt{\frac{\rho_{}}{\upsilon_{e}m_{k}}}l_{k}^{}$$

The Kuhn segment length is the virtual statistical step length for which a polymer chain can be described as a freely jointed chain. The Kuhn segment length is also a measure for the free space mechanical stiffness of the chain, expressed by the persistence length, which is equal to *l_k_*/2 [14]. Combination of Equation (1) with Equation (2) shows that Γ ~ $\upsilon_{e}d_{e}$ scales as $\upsilon_{e}{}^{1/2}$. Σ*_e_* represents the axial density i.e., the number of load bearing chains crossing a mathematical surface which is topologically related to *ν_e_* as Σ*_e_* = 1/2*ν_e_d_e_*.

**Figure 5:**
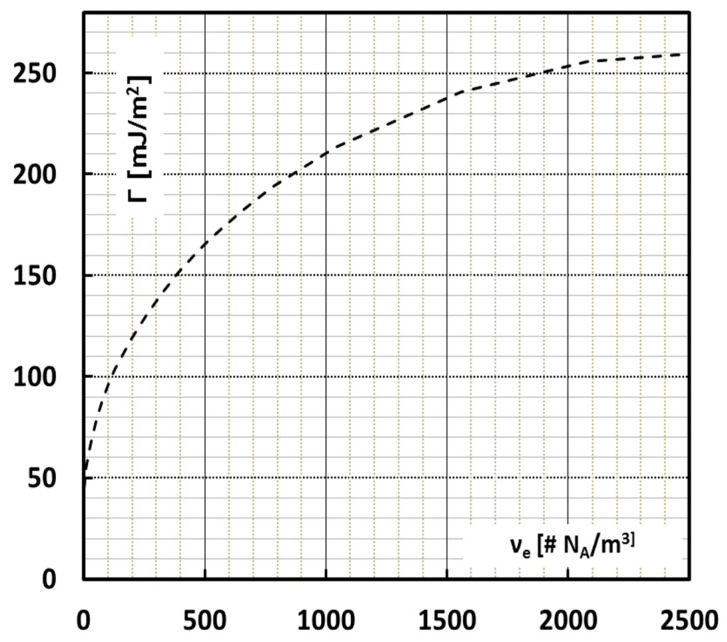
Surface energy Γ as a function of the effective network node density expressed in multiples of Avogadro’s number assuming the Porod worm chain model of Equation (6).

Equation (2) is, of course, only valid when the chain segment between nodes is sufficiently long to allow for Gaussian statistics to be applied, which is usually the case in polyolefin melts and rubbers. However, with increasing network densities, the number of Kuhn segments may require another statistics to calculate *d_e_*. For shorter chain segments, the Porod worm chain model should be adopted [13]:

(4) $$d_{e}=\sqrt{l_{k}L_{e}\left(1-\left(\frac{l_{k}}{2L_{e}}\right)\right)\left(1-e^{\left(-\frac{2L_{e}}{l_{k}}\right)}\right)}$$

where *L_e_* is the contour length of the *M_e_* segment. Clearly Equation (4) reduces to Equation (3) for larger *n_L_*. Therefore, in this work, the Porod model is adopted for all calculations of *d_e_*, irrespective of the network density up to a network with shear modulus of 5 MPa, which would make *d_e_* smaller than the Kuhn length, which is physically impossible. Figure 5 shows the surface energy as a function of the available network density according to Equation (1) taking into account the Porod statistics.

The nodal network with density $\nu_{e}$ can be described by its shear modulus *G*′ following rubber elastic theory:

(5) $$G^{\prime }=A\upsilon_{e}RT$$

where *A* is the network topology factor which we assume to be unity following the work of Fetters [15]. Equation (5) describes a system of fixed nodes connected by Gaussian chains (i.e., a cross linked rubber) but it is also used to describe time-dependent networks with both fixed and transient nodes [16]. This rubber elastic description assumes that there is a proportionality of the network shear modulus with temperature and we know that this is not the case. The shear modulus of an uncrosslinked melt entanglement network and the strain hardening modulus in the solid state both decrease with *T* and increase with the strain rate which indicates that this network response is mainly enthalpic [17,18] and probably mainly caused by segmental and monomeric friction. Notwithstanding this enthalpic nature, Van Melick et al. found that in glassy polymers there is still a proportionality of the strain hardening modulus with the melt entanglement density with a negative linear T dependence, which scales with the yield stress [19].

The craze microstructure at the time of failure in a slow crack growth process and the time of failure at a given stress in such an experiment have recently been shown to be determined by the strain hardening modulus, determined above the alfa transition [20]. A practical way of representing the surface energy is therefore via its proportionality with the network shear modulus *G*′:

(6) $$\Gamma =\gamma +\frac{1}{4}\sqrt{\frac{\rho_{am}}{ARTm_{k}}}\sqrt{G\prime }$$

where *ρ_am_* is the amorphous density and *m_k_* is the mass of one Kuhn segment. Notice that when the shear modulus vanishes, the surface energy reduces to the Van der Waals value *γ* indicative of disentanglement crazing at low strain rates and high *T* [12].

Physical validity of this surface energy concept can be derived from comparison with available experiments and estimations. In Kramer and Berger’s work on craze propagation in amorphous polymers, the value for Γ is taken from the rubber plateau modulus [12]. This is justified because the mechanically activated continuum at the craze tip is actually in a mechanically liquefied state, comparable to the melt. For polystyrene, this leads to a total surface energy of 0.080 J/m^2^, assuming a plateau modulus of 0.20 MPa and a Van der Waals surface energy of 0.04 J/m^2^. In that same work, also a strain rate or a temperature dependence of the network density and hence of Γ is assumed in order to describe the transition from scission crazing to disentanglement crazing as a thermally activated process of chain slip.

### 4.2. The Solid State Surface Energy in Impact Conditions

For the description of the surface energy in the semi-crystalline solid state at short time scales and low T we need to know the number of chains that can be loaded effectively and broken in the course of the surface creation process. There are some obvious Gedankenexperiment-like upper limits to that value; for example, if one were to fracture all chains aligned in a crystal one would have to muster 1.57 J/m^2^ for an orthorhombic Polyethylene crystal and for Polypropylene in the monoclinic α phase this would be 0.840 J/m^2^. Another upper bound estimate comes from following Huang and Brown’s argument [21] that in an isotropical amorphous phase, geometrically, one third of the molecular stems are sufficiently oriented and assuming that no slip is possible for all these chains, 30% of that upper bound crystalline surface energy is available, i.e., 253 mJ/m^2^ for PP. A lower bound estimate finally would be to take the entanglement density at the rubber plateau *G*′ = *G*_N_^0^ which leads to a value of 0.107 J/m^2^.

There are not many experiments that try to relate the fracture stress to the available load-bearing surface (potential surface energy). This surface fraction of tie molecules in polyethylene has been estimated by Brown and Ward, in their famous fracture experiment at 90 K [22] on polyethylenes. They find up to 23% of surface to be effectively load bearing during the fracture test, which leads us to an estimate of 0.361 J/m^2^, in PE. For PP the equivalent estimate would be 0.196 J/ m^2^. This compares well with the above estimates.

An experimental attempt to estimate the surface energy from Griffith’s fracture mechanics [1] approach leads to surface energies, three to four orders of magnitude too large. When one assumes that the critical stress intensity K_1c_ of iPP lays typically around 3.3 MPam^1/2^, Griffith’s theory leads to a surface energy of 5 kJ/m^2^. This huge discrepancy is attributed to volume plasticity contributions, typical for a fracture mechanics test, which was exactly what Brown and Ward tried to avoid in their experiment [22].

It is clear that the surface energy needed to fracture a semicrystalline polymer will be determined by the number of chains that can be loaded and broken in an impact experiment and that number will for impact conditions be higher than the rubber plateau network. We surmise that a valid measure for that number of chains, Σ*_e_* is the solid state strain-hardening modulus [5], following the work of Haward [23,24] and Duffo et al. [25]. For longer time scales, like in slow crack growth processes, the contribution of the crystal phase will be transient and the network will be limited to a number in the neighborhood of the plateau modulus, with typical shear moduli of the order of 0.5–1.5 MPa. For shorter time-scale processes such as impact at low temperatures, the crystalline phase will function as a robust restraint for the chain segments to slip and virtually all the available nodes will act as fixed nodes. The strain-hardening modulus of solid iPP in the α phase varies from 0.46 MPa at 110 °C to 4.6 MPa at room temperature [25]. This would indicate that, going from slow (high *T*) to fast deformation (low *T*) processes, the number of chains loaded may increase a decade or even more. Of course one should be aware that in this transition the material goes from a rubber-like state to a heterogeneous semicrystalline material with some strain localisation caused by the crystals, both in the isotropic regime around yield as in the strain hardening. The working hypothesis we adopt here is that, notwithstanding varying tractability of the crystals, the material can still be considered to form a rubber-like continuum where the strain-hardening response is a measure for the number of load bearing molecular stems at the temperature and the strain rate of the experiment. The surface energies calculated from the assumption that the strain hardening modulus is a direct measure of the number load bearing chains between *G_p_* = 0.46 and 4.6 MPa, range from 0.107 to 0.205 J/m^2^ where it was taken into account that there is a physical limit to the network density when the chain segment becomes equal to the Kuhn length. In our set of experiments, Γ then typically lies between 0.170 and 0.205.

The order of magnitude difference in the network density going to higher strain rates and lower temperature can also be rationalized by looking at the ratio between the number of slipping network links and the number of fixed links in a normal tensile experiment, as calculated from a fit with a slip link model [26,27,28] which is typically a factor of 10.

Conclusion of this section is that depending on strain rate and temperature, the network density may vary and consequently the surface energy will also vary leading to a reformulation of Equation (1) to include the rate and temperature dependence:

(7) $$\Gamma \left(T,\overset{•}{\varepsilon }\right)=\gamma +\frac{1}{4}d_{e}(T,\overset{•}{\varepsilon })\upsilon_{e}(T,\overset{•}{\varepsilon })U_{b}$$

### 4.3. Cavitation, the Lazerri–Bucknall Energy Criterion

Provided that the rubber phase is able to build up sufficient elastic energy, the negative hydrostatic stress will cause rubber particles to void prior to shear deformation of the PP matrix, if the yield stress of the remaining ligament happens to be lower than the brittle fracture stress. This can only happen above the glass temperature (*T_g_*) of the rubber phase; otherwise, the rubber phase will not localize the strain nor build up elastic energy. This *T_g_* has of course to be adapted for the strain rate of the experiment, for example, the *T_g_* at 1 Hz (the standard frequency of a dynamic mechanical thermal analysis (DMTA) test) of the rubber phase in rubber-toughened PP lies around −60 °C. In Izod impact tests the rate is equal to a frequency of 3000 Hz, three decades faster than the DMTA *T_g_* measurement, in a standard time-temperature equivalence shift (7.8 K/decade), and this implies that rubber particle cavitation is limited to about −36 °C in impact. This generally fits the lowest observed ductile temperatures in rubber-toughened PP well [9].

A lower brittle–ductile transition temperature could be reached if a lower *T_g_* of the rubber phase is achieved. The lowest possible limit would be achieved by the glass temperature of silane rubbers which is around −125 °C in a DMTA measurement, resulting in about −100 °C under impact conditions.

### 4.4. The Energy Balance Description within the Rubber Particle

The critical size of the rubber particle *d_c_* for cavitation to occur can be calculated from the cavitation energy balance equation as formulated by Lazzeri and Bucknall [29]. The energy of a cavitated rubber particle:

(8) $$U=\frac{2}{3}\pi R^{3}K_{r}{\left(\Delta -\frac{r^{3}}{R^{3}}\right)}^{2}+4\pi r^{2}\Gamma +2\pi r^{3}G\rho F(\lambda_{f})$$

where Δ is the volume strain of the rubber particle, *R* is the particle radius and *r* the cavity radius. *K_r_* is the bulk modulus of the rubber in the particle. Γ represents the energy required to create surface as defined in Equation (4), *G* is the shear modulus of the rubber. *ρ* is the density ratio of the rubber phase prior and after cavitation which can to good approximation be considered to be 1. *F*(*λ_f_*) is a strain function at biaxial failure strain which is typically of the order of unity [29]. Prior to cavitation (*r* = 0) there is only the elastic volume strain energy built up. Equation (7) allows the contributions prior to and after cavitation to be calculated. As stated by Lazzeri and Bucknall the driving force of the cavitation will be lowering of the total energy to a minimum i.e., $\frac{\partial U}{\partial r}=0$.

They found that minimum to be close to $\Delta =\frac{r^{3}}{R^{3}}$, i.e., complete vanishing of the accumulated strain energy. One can further assume that the third term in Equation (7) will be balanced by a quasi equal traction on the surrounding matrix [30], so that the energy balance within the rubber particle before and after cavitation comes down to equating the two first terms of Equation (7). Switching from radia to diameters *d*_0_ = 2R and *d_i_* = 2*r* and using the relation *d_i_* = Δ^1/3^*d*_0_, the condition for cavitation to occur becomes:

(9) $$U_{tot}=U_{\varepsilon }+U_{\Gamma }=-\frac{\pi }{12}K_{r}\Delta^{2}d_{0}^{3}+\Gamma \pi \Delta^{\frac{2}{3}}d_{0}^{2}<0$$

where *U_e_* and *U*_Γ_ are the elastic and the surface energy respectively. *K_r_* is the bulk modulus of the rubber particle, Δ is the volume strain, *d*_0_ is the initial rubber particle size, and Γ is the surface energy as described by Kramer and Berger’s equation shown in Equation (2).

The energy criterion as shown in Equation (9) has also been formulated by Dompas and Groeninckx [31].

The critical condition *U_tot_* = 0 yields the critical particle diameter from Equation (9) combined with Equation (6):

(10) $$\begin{array}{l} d_{0}^{c}=\frac{12\Gamma (\dot{\varepsilon },T)}{K_{r}\Delta^{\frac{4}{3}}}\\ =\frac{12\left(\gamma +\frac{1}{4}\sqrt{\frac{\rho_{am}}{ARTm_{k}}}\right)\sqrt{G\prime ((\dot{\varepsilon },T))}}{K_{r}\Delta^{\frac{4}{3}}} \end{array}$$

**Figure 6:**
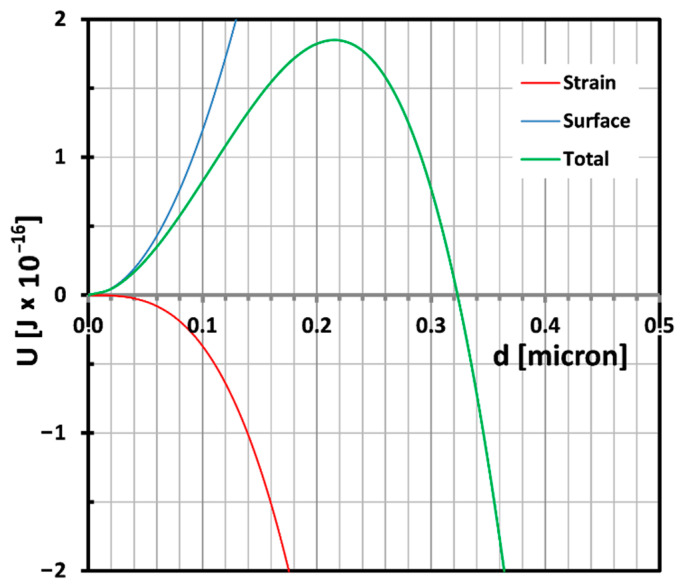
The balance of strain and surface energy in a rubber particle as a function of particle diameter for *K_r_* = 2.5 GPa and *G* = 0.4 MPa. The volume strain is the critical value proposed by Dijkstra et al. i.e., 0.75% [32]. The critical particle diameter in this case is then 330 nm.

This equation shows clearly that the critical particle size depends on the ratio of the shear modulus over the bulk modulus. If easy cavitation is the goal, a high bulk modulus and a low shear modulus are desirable. Typical values for shear moduli and bulk moduli range around 0.5–1.5 MPa and 2–3 GPa respectively. A typical candidate for good cavitation properties in that respect should be a polydimethyl siloxane (PDMS) rubber. The balance described in Equation (9) is plotted in Figure 6 for a shear modulus of 0.4 MPa and a bulk modulus of 2.5 GPa assuming a volume strain of 0.75% which is a typical value for cavitation occurrence [32].

**Figure 7:**
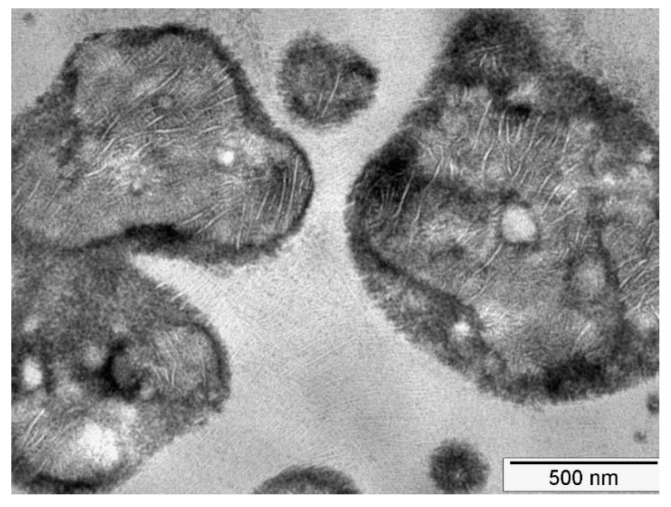
Transmission electron microscopy (TEM) image of the typical particle morphology in the present series of reactor copolymers. Cross-hatched lamellae are visible in the matrix phase. The dark areas are EPR rubber. The light grey blobs inside the particles are LLDPE like polyethylene (PE) and the whiter parts inside the particle are matrix occlusions. (Courtesy of Peter Neuteboom (Sabic Geleen)).

In the present kind of impact-resistant EPR copolymers, the rubber particles are heterogeneous in nature. They are composed of areas with varying composition ranging from PE-rich LLDPE-like areas (featuring the typical morphology of Linear Low Density Polyethylene) to PP-rich occlusions, as shown in the transmission electron microscopy (TEM) image in Figure 7.

Such a morphology with large differences in local modulus will lead to strain localization in the EPR domains. Because of the low surface energy, these EPR areas will cavitate preferentially as has been shown by means of Scanning Transmission Electron Microscopy computer tomography [33]. In these images it is also easily seen that the PE and the PP domains do not feature cavitation which is also to be expected in view of the tenfold higher surface energy of these areas. It is assumed that the stored elastic energy that drives the cavitation in these particles will be identical to the energy stored in a homogenous particle.

**Figure 8:**
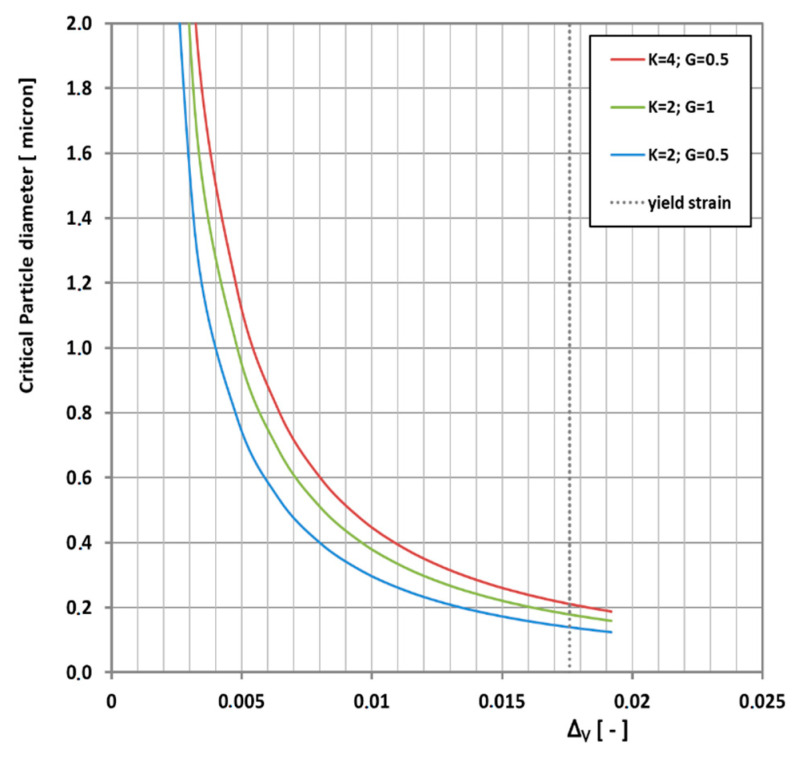
Critical particle size for cavitation according to Equation (10) for a collection of bulk and shear moduli. The grey vertical dotted line is the matrix yield strain, beyond which there is no hydrostatic cavitation stress.

Equation (10) is shown in Figure 8 for some plausible combinations of bulk and shear moduli.

Increasing the bulk modulus (or density) decreases the critical particle size and increasing the shear modulus increases the critical particle size. Furthermore, it is physically impossible to go beyond yield strain, because when the matrix yields it cannot convey elastic stress to the rubber particles anymore. Particles smaller than the critical particle size will deform affinely with the matrix and will not cavitate. This limit is indicated by the vertical dotted line in Figure 8.

### 4.5. The Critical Matrix Ligament Thickness for Ductile Failure

After cavitation, the failure mechanism depends on the properties of the matrix ligaments. Two major factors need to be studied: the glass temperature of the matrix PP, and the ligament thickness. An elegant way of calculating the critical matrix ligament thickness for ductile failure is the Van der Sanden, Meier and Tervoort (VMT) criterion [11]:

(11) $$ID_{c}\leq \frac{6\Gamma E_{m}}{\lambda_{\max}\sigma_{ym}^{2}}$$

This is derived from the energy balance between the available elastic energy within the deforming ligament, represented by the yield stress, and the energy required to create a brittle fracture, represented by Γ. *E_m_* is the matrix tensile modulus, $\sigma_{ym}$ is the yield stress of the PP matrix and *λ*_max_ is the maximum draw ratio of the matrix. The matrix *T_g_* as it will be shown later influences mainly *σ_ym_*, but also Γ, *E_m_* and even *λ*_max._ Assuming literature melt entanglement densities and normal tensile test conditions at room temperature, the critical ligament size in rubber-toughened PP to achieve toughness is of the order of 2 µm.

### 4.6. Maximum Drawability of the Ligament

Equation (10) features the drawability of the ligament *λ*_max_. This can be easily calculated from the number of Kuhn segments between nodes defined by Equation (3):

(12) $$\lambda_{\max}=\sqrt{n_{{}_{L}}}$$

To this theoretical value of *λ*_max_ one should also apply Kramer’s empirical stretchability limit [12]. This limitation of stretchability, specifies that the maximum draw ratio is limited to 60% of *λ*_max_. This is generally attributed to network imperfection and seems to be applicable to most polymers.

### 4.7. The Rate and Temperature Dependence of the Network and its Physical Limits

Now that we have realized that the surface energy is determined by the available rate and temperature dependent network density i.e., the available assembly of effective tie molecules, it is necessary to calculate this density. As stated above in Section 4.2 we assume that this network density is described by the shear modulus of the perceived rubbery response [5], the strain-hardening modulus *G_p_*.

The temperature dependence of the strain hardening modulus of isotactic PP has been measured very accurately [25].

**Figure 9:**
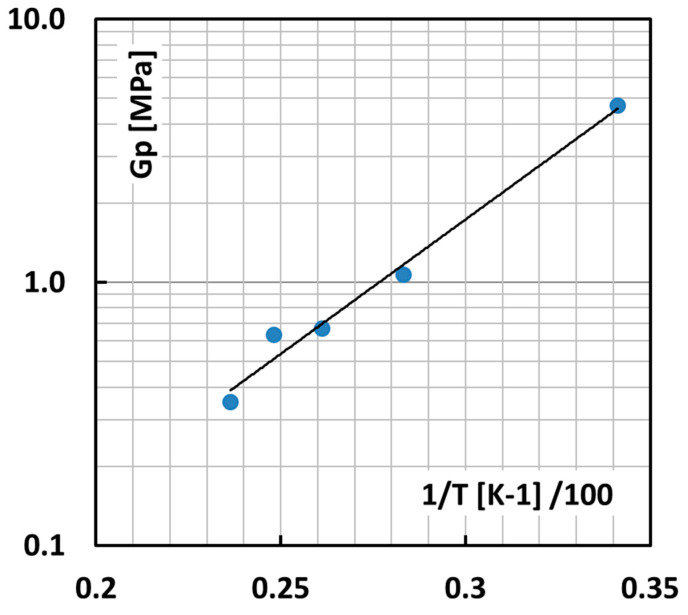
Strain-hardening modulus, representing the network density by Equation (5), as a function of temperature, after our own Neo Hookean fits on the work of Duffo et al. [25]. The abscissa is the reciprocal absolute temperature multiplied by 100 for convenience.

This leaves us with a useful experimental range for the T dependence of the apparent node density in the solid state between room temperature (RT) and 150 °C as shown in Figure 9 as a log *G*′ versus 1/T plot.

The straight extrapolation of the line in Figure 9 to low T leads eventually to non-physically dense networks with shear moduli far beyond 5 MPa, meaning contour lengths tend to become smaller than the Kuhn length. In this case, it is assumed that the contour length is at least equal to the Kuhn length and consequently *n_L_* = 1 and *λ*_max_ = 1. The surface energy then reaches a maximum of a very acceptable 0.205 J/m^2^.

### 4.8. Rate and Temperature Dependence of the Matrix Yield Stress

The iPP matrix typically has a *T_g_* of 273 K and it is possible that the amorphous PP rubber is in its glassy state at the time scale of a typical impact test. Therefore, it is important to know the influence of the bulk *T_g_* on the yield stress of iPP. To this end, the literature was screened for such data and some valid and useful plane stress compression measurements for a very wide strain rate range from Okereke et al. [34] was found. Since the iPP matrix ligaments are tensile loaded in the direction of the applied stress, the compression tests of Okereke et al. need to renormalized to tensile data. Such tensile data were obtained by Van Erp et al. [35]. As usual the tensile values are lower. This is attributed to two major causes; the first is the opposite sign of the hydrostatic stress in a compression test as compared to a tensile test. This has already been investigated by Mears et al. [36] who investigated the effect of hydrostatic pressure on both PE and iPP. Kanters et al. [37] demonstrated how large the hydrostatic effect can be and explains that it can depend on the crystallinity and processing conditions. A second important reason is the friction with the compression plates, which is expected to increase linearly with strain rate, i.e., the ratio between the compression and the tensile value of the yield stress must be constant. However, in sound experiments this friction contribution should be reduced to negligible values, especially at the yield stress where the strain is still quite low.

**Figure 10:**
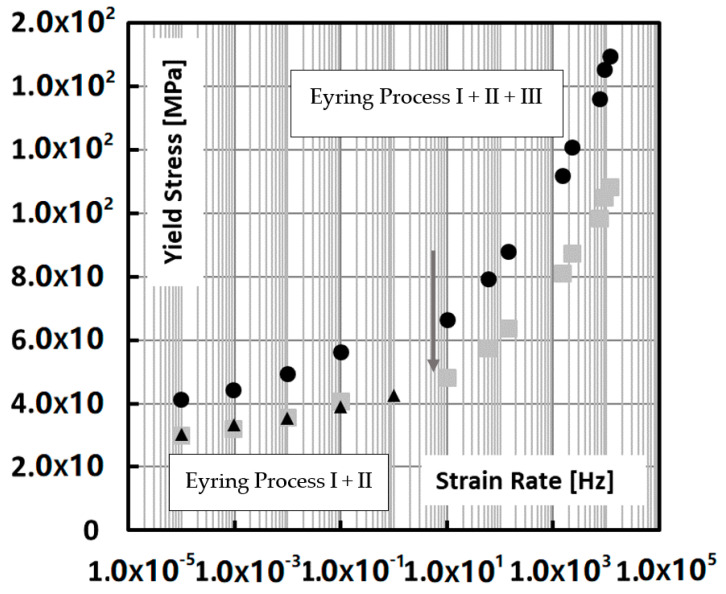
Strain rate dependence of the yield stress of isotactic PP. The black dots represent the compression measurements by Okereke et al. [34], the large grey squares is the same data set corrected for tensile axial load and friction, based on data measured by Van Erp et al. [35] which are plotted in black triangles. The grey arrow indicates the assumed onset of *T_g_*. Also the Eyring processes mentioned in the text are indicated.

Neglecting friction contributions, an average ratio between compression yield stress and tensile yield stress of 1.38 was observed and applied to transform the compression data to tensile data as it is shown in Figure 10. This way an estimate of the high strain rate tensile behavior of an iPP matrix ligament is obtained. A note of caution may be necessary here, since our renormalisation implicitly assumes that the processing conditions for both data sets are comparable, which might not have been the case.

**Figure 11:**
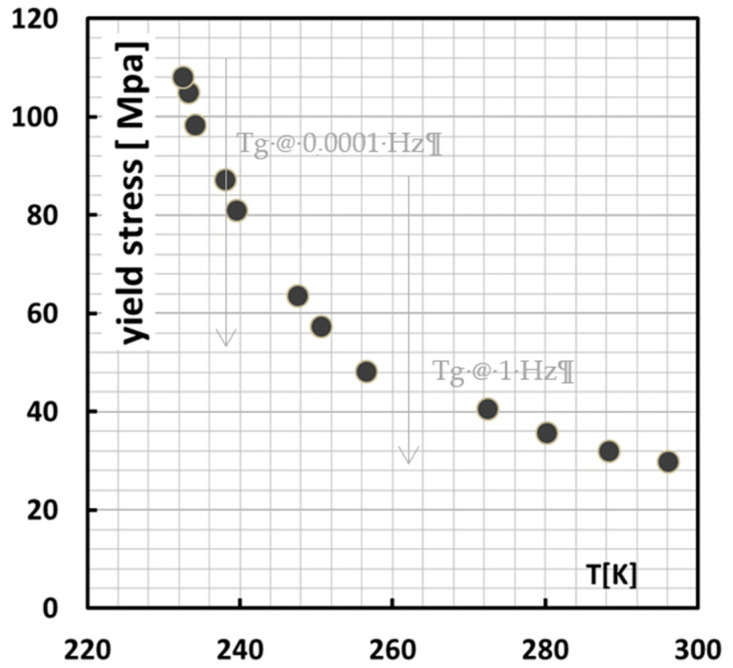
T dependence of yield stress at a strain rate of 0.0001 Hz around *T_g_* as calculated from the data in Figure 3, assuming a t,T equivalence of 7.8 K/decade. The arrows indicate *T_g_* at 1 Hz, and at 0.0001 Hz.

Assuming a t,T shift factor of 7.8 K/decade, which is quite common for all t,T shifts in polymers [38] for creep and yield, this rate dependence can be calculated into a T dependence. The reference rate is taken at the lowest rate, i.e., 0.0001 Hz as is shown in Figure 11. From RT, for example, it would then take, starting at the lowest test rate, ~3–4 decades to attain *T_g_*. This would situate the *T_g_* at around 1–10 Hz, which is what the grey arrow in Figure 11 suggests, where indeed an extra rate-dependent regime kicks in.

What Figure 10 and Figure 11 also suggest is that this strain rate/temperature dependence beyond *T_g_* is not typically an Eyring process, which would imply a straight line in log (t), but rather a curved Williams Landel Ferry (WLF)-type dependence [38].

At this point, it is also meaningful to look closer into the work of Van Erp who analysed the rate dependence of polypropylenes in terms of the Ree–Eyring activated flow model [35]. Generally two Eyring rate regimes are encountered, i.e., process I (low strain rates, high temperatures) and process II that kicks in on top of process I at increased strain rates and lower temperatures. In the range of the Okereke data, there appears to be a third relaxation process on top of process II, most probably caused by the vitrification of the amorphous phase. This third relaxation process has also been confirmed by Caelers et al. in their research on the differences in the creep response of iPP polymorphs [39].

The present data for the constitutive behavior of isotactic polypropylene shown in Figure 10 and Figure 11 are used to calculate the yield stress by inter- or extrapolation of the properly temperature or rate shifted data. However, there is a hard physical limit to this extrapolation in that the estimated yield stress should never exceed the true fracture stress which is typically 250 MPa. This limit is set as a threshold value in the in the calculation of the critical ligaments thickness.

### 4.9. The Rate and Temperature-Dependent Van der Sanden, Meijer and Tervoort (VMT) Equation for Impact

With the yield stress, drawability and the surface energy dependency on rate and temperature made explicit we are able to fill in the rate and temperature-dependent VMT equation:

(13) $$ID_{c}\left(T,\overset{•}{\varepsilon }\right)=\frac{6\left(\gamma +1/4d_{e}(T,\overset{•}{\varepsilon })U_{b}\upsilon_{e}(T,\overset{•}{\varepsilon })\right)}{\lambda_{\max}(\upsilon_{e}(T,\overset{•}{\varepsilon )})\sigma_{ym}^{2}(T,\overset{•}{\varepsilon })}$$

**Figure 12:**
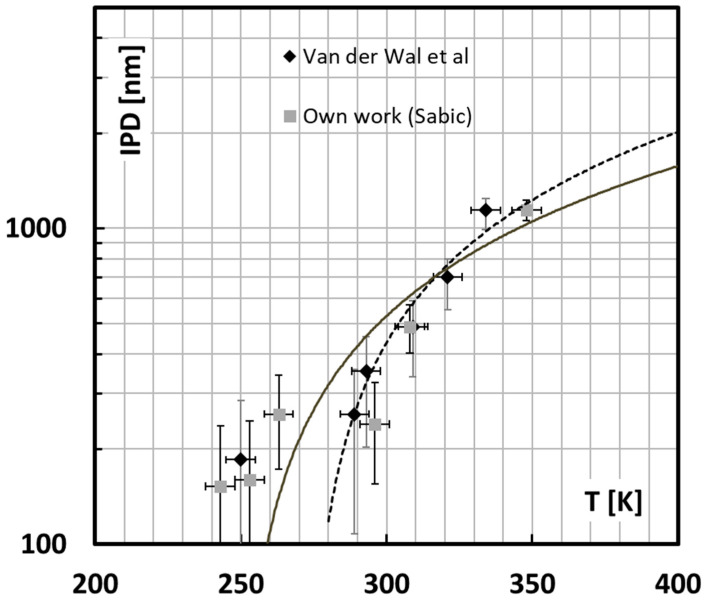
Comparison between experimental data from SABIC (grey squares) and Van der Wal et al. (black diamonds) represented on average by the solid line and the theoretical prediction (dotted line) using Equation (13).

**Figure 13:**
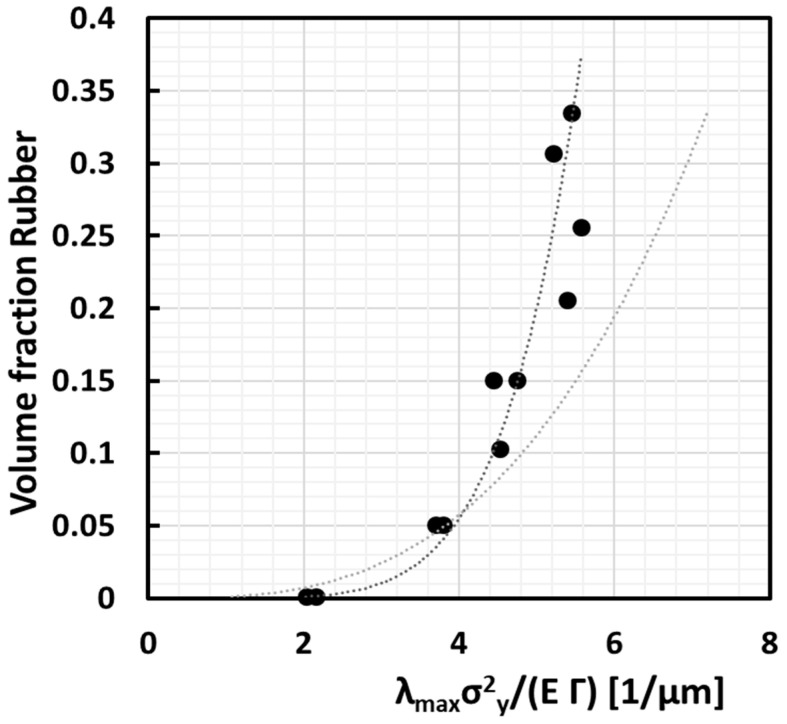
Proportionality of volume fraction of rubber with critical ligament size in arbitrary units, according to the model in this paper, assuming cubic stacking of monodisperse rubber particles with size 1.44 µm. Black curve is the fit with experimental data, which scales with exponent 5.3; grey curve illustrates the third power scaling.

It becomes now possible to use Equation (13), respecting the physical limits of the network density and the yield stress to interpret the available experimental data from Van der Wal’s dataset [6,7,8,9,10] and from our own work, shown in Figure 12. For this calculation it is assumed that the rubber particles are monodisperse and stacked according to a cubic stacking scheme so that all matrix ligaments have the same size. One can then plot the critical interparticle distance as a function of the brittle ductile transition temperature and compare it to these experiments as shown in Figure 13.

In view of the relative simplicity of the model and the assumptions on the morphological analysis, this is a very satisfying result. The overall trend of the observations is followed albeit the model predicts a much smaller critical interparticle distance than the observations reveal for high rubber content. This is because in a real blend the heterogeneous spatial distribution allows much smaller interparticle distances than the average calculated from the blend morphology especially at high rubber content.

In a next stage it becomes plausible to investigate possible gains in toughness from adapting the morphology according to this simplified model.

### 4.10. Effectivity of Rubber Dispersion

Combination of the Lazerri–Bucknall energy criterion of Equation (9), assuming a cubic stacking of monodisperse particles from Equation (1), with the VMT model of Equation (12) taking <*d*> = *d*_0_*^c^* allows to single out the volume fraction of rubber *φ_r_* written to first order as a proportionality with $\frac{\lambda_{\max}{\left(\sigma_{y}^{m}\right)}^{2}}{E_{m}\Gamma_{m}}$, which is proportional with the inverse critical ligament thickness *ID*_c_.

(14) $$\begin{array}{l} \varphi_{r}=\frac{\pi }{6}{\left[\frac{\Gamma_{m}E_{m}(\dot{\varepsilon },T)K_{r}\Delta^{\frac{4}{3}}}{2\Gamma_{r}(\dot{\varepsilon },T)\lambda_{\max}^{m}(\dot{\varepsilon },T){\left(\sigma_{y}^{m}(\dot{\varepsilon },T)\right)}^{2}}+1\right]}^{-3}\propto {\left[\frac{\lambda_{\max}(\dot{\varepsilon },T){\left(\sigma_{y}^{m}(\dot{\varepsilon },T)\right)}^{2}}{E_{m}(\dot{\varepsilon },T)\Gamma_{m}(\dot{\varepsilon },T)}\right]}^{3}\\ \varphi_{r}\propto {\left[ID_{C}\right]}^{3} \end{array}$$

where it is assumed that the volume expansion at cavitation, the bulk modulus and the surface energy of the rubber are constant observables. Figure 13 shows this ideal proportionality (grey curve) compared with the experimental data (black curve). The final fit looks more than good with the exception that the black curve increases with a power higher than 3, i.e., 5.3. This may be attributed to the fact that actually a lot more rubber is needed whenever the distribution deviates from the ideal monodisperse and homogeneous distribution. The theoretical achievable limit for a perfect dispersion should follow a third power ideally. It is clear from Figure 13 that rubber can probably be distributed much more effectively to achieve a given average ligament size. This would allow achieving a much better toughness/stiffness balance because a rubber content of evenly distributed monodisperse rubber particles of 15 vol % could then already lead to the same brittle ductile temperature as the 30 vol % polydisperse case.

## 5. Conclusions

The VMT model for describing the critical ligament thickness in rubber-toughened polymers has been extended to use for low *T*, impact conditions. To that end the rate dependencies of the constitutive behaviour of the PP matrix as known from literature are implemented, the rate dependency of the surface energy is extrapolated with the rate increase of the network density and this is justified by comparison with Brown–Ward’s experiments on the fracture stress of PE. The extrapolation to impact conditions is compared with data from literature and confirmed.

The critical particle size model of Lazerri and Bucknall is combined with the adapted VMT model. Comparison with experimental data on brittle ductile transitions confirm the combined model in that the inverse critical ligament size scales to the >5th power with the rubber volume fraction.

This scaling indicates the general inefficiency of the particle size distribution and its homogeneity i.e., an ideal stacking should show a third power, so a lot of toughening efficiency of the rubber phase is to be gained by making the rubber stacking more regular.

## Data Availability

The data and calculations are kept at SABIC T&I in the KMP database.
